# Analysis of malnutrition factors for inpatients with chronic kidney disease

**DOI:** 10.3389/fnut.2022.1002498

**Published:** 2023-01-06

**Authors:** Wei-zhen Xi, Chen Wu, Ya-li Liang, Ling-Ling Wang, Yu-han Cao

**Affiliations:** Kidney Internal Medicine, The First Affiliated Hospital of Wannan Medical College, Wuhu Anhui, China

**Keywords:** chronic kidney disease, malnutrition, SGA nutritional assessment, public health, treatment

## Abstract

**Objective:**

Malnutrition is a common complication of Chronic Kidney Disease (CKD), and it is the risk factor of CKD prognosis. This study aim to evaluate the nutritional status of inpatients with CKD by using the Subjective Global Assessment (SGA), and to analyze the related factors of malnutrition; and to provide effective reference for early detection of malnutrition status in patients with CKD and timely nutrition intervention.

**Methods:**

A total of 426 patients (238 male patients, 188 female patients) aged 62.62 ± 14.61 and 61.14 ± 14.82, respectively admitted to the Nephrology Department of Wannan Medical College from February 2020 to December 2020 were selected and included in to this study by convenience sampling. 426 patients with CKD were evaluated by SGA. Human body weight, hemoglobin (Hb), total protein (TP), albumin (ALB), pre-albumin (PA), qualitative analysis of urinary protein and other laboratory indexes were collected and measured. The correlation between malnutrition and age, education, gender, diet, CKD stage and other factors was analyzed by spearman correlation analysis.

**Results:**

The incidence of malnutrition was 85.7% among 426 patients with CKD. Gender, age, education level, CKD stage, diabetes mellitus, weight loss and reduced food intake were related to SGA nutritional assessment (*P* < 0.05). The expression levels of ALB, PA and Hb in the malnutrition group were significantly lower than those in the normal group (*P* < 0.05). The degree of malnutrition in CKD patients was significant negatively correlated with the expression levels of ALB (*r* = −0.188), PA *(r* = −0.262) and Hb (*r* = −0.176) (*P* < 0.05). The multivariate Logistic regression analysis model showed that female (OR = 2.155), ≥60 years old (OR = 7.671), weight loss (OR = 10.691), reduced food intake (OR = 28.953), moderate and severe serum ALB expression (OR = 3.391 and 8.326) were risk factors for malnutrition in patients with CKD (*P* < 0.05). Malnutrition was correlated with the results of qualitative examination of urinary protein (*r* = 0.268, *P* < 0.05).

**Conclusion:**

Gender, age, weight loss, reduced food intake, serum ALB expression were independently associated with malnutrition in patients with chronic kidney disease, Hence, the medical staff should take timely and effective nutrition intervention for the patients with malnutrition, delay the renal function damage of patients with CKD and improve the quality of life of patients. Inpatients with CKD, especially women, should increase their dietary intake, maintain normal weight and improve their nutritional status.

## Introduction

Chronic Kidney Disease (CKD) refers to the chronic renal structure and dysfunction (>3 months) caused by various reasons, including normal and abnormal pathological damage of Glomerular Filtration Rate (GFR), abnormal blood or urine components and imaging examination, or GFR decline for unknown reasons (GFR <60 ml/min) for more than 3 months. The prevalence of chronic kidney disease in the world is about 14.3% (1), and that of CKD in China is about 10.8% (2). CKD has become an important public health problem which has a high prevalence, poor prognosis and expensive medical expenses.

CKD is a complex disease. There are many important factors which affects the status and progression of CKD patients. Old ages and low glomerular filtration rate are more probably to develop geriatric syndromes and malnutrition (3). Malnutrition is a common complication of CKD, and it is the risk factor of CKD occurrence (3), progress (4), cardiovascular events and death (5). The cardiovascular events and death risks of CKD patients increased significantly with the decrease of renal function (6).

The prevalence of malnutrition in CKD patients in China is 22.5–58.5%, the prevalence of malnutrition in peritoneal dialysis patients is 11.7–47.8%, and that of hemodialysis patients is 30.0–66.7% (7). Therefore, it is very important to pay attention to the nutrition of CKD patients and to carry out nutrition treatment throughout the CKD treatment process, which is of great significance to improve the overall diagnosis and treatment level of CKD, delay the progress of disease, improve the prognosis of patients and reduce the medical expenses.

## Materials and methods

### Clinical data

A total of 435 patients admitted to the Nephrology Department of Wannan Medical College from January 2020 to December 2020 were selected by convenience sampling. Inclusion criteria: ① Patients diagnosed with CKD according to the 2006 NKF-K/DOQI standard; ② Age ≥16 years old; ③ Patients willing to accept the SGA nutritional evaluation; ④ Patients with albumin (ALB), pre-albumin (PA) and hemoglobin (Hb) indexes were checked by blood sampling during hospitalization. Exclusion criteria: ① Patients <16 years old; ② Complicated infection (those infections associated with fevers, stones, sepsis or involving the kidneys), hyperthyroidism, tumor and other diseases in the last 3 months may affect the nutritional status; ③ Surgical treatment or other medical operations within the last 3 months may affect the nutritional status; ④ Refused to participate. Six patients were excluded from this study due to meeting the exclusion criteria complicated infection and 3 patients refused to participate in this study. Finally, a total 426 patients were included in this study and their data was collected and analyzed.

### Methods

#### Data collection

By unified training investigators to conform to the standard of hospitalized patients with CKD for data collection, including gender, age, education, marital status, payment, height, weight and whether the risk of high blood pressure, diabetes, gout, coronary heart disease, cerebral infarction, and CKD stage, whether long-term hemodialysis.

#### Biochemical indicators

Fast-fasting blood samples were collected on the second day after hospitalization, total protein (TP), albumin (ALB), prealbumin (PA), hemoglobin (Hb) and qualitative analysis of urinary protein were checked and measured by hospital laboratory.

#### SGA nutritional evaluation

The SGA was used to evaluate the nutritional status of 426 patients with CKD (8). The scale is a universal tool for assessing clinical nutritional status. It includes history (changes in weight, changes in food intake, gastrointestinal symptoms, changes in mobility, metabolic demands in disease state) and physical examination (loss of subcutaneous fat). The SGA evaluation was questioned and collected by researchers.

The SGA scores were: A = Normal, B = mild and moderate malnutrition, and C = severe malnutrition. The SGA is used by a trained dietitian to assess the patient's nutrition.

#### Quality control and data entry

The participants in the survey were all medical staff from the nephrology department and nutrition department of our hospital. They participated in the unified training, conducted nutrition assessment and questionnaire survey together.

#### Statistical methods

Collected data were double-entered and double-verified using Epidata version 3.1. Statistical Package for Social Sciences (version 21.0; SPSS) was used to analysis. Quantitative data that obeyed a normal distribution were expressed as mean ± standard deviation (X ± S), and comparisons between groups were performed using the *t*-test. Qualitative data were expressed as frequency (n) together with percentage (%), and Chi-Square (χ^2^) test was used for comparison between groups. Spearman rank correlation was used to analyze the correlation between the two variables, and ordinal logistic regression was used to analyze the influencing factors of malnutrition. A *P-*value of < 0.05 was considered statistically significant.

## Results

### Relationship between general clinical characteristics of CKD patients and SGA nutritional assessment

**Figure 1:**
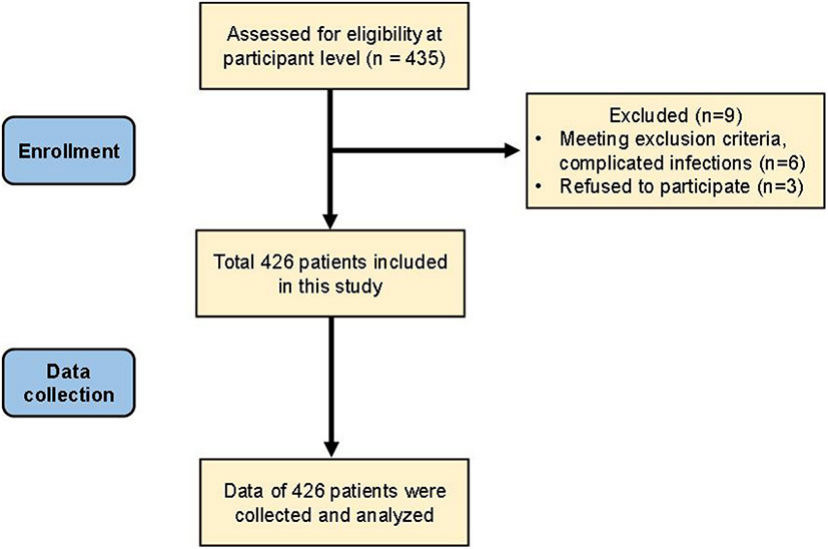
Flow chart of this study design.

In this study, a total of 435 patients admitted to the Nephrology Department of Wannan Medical College from January 2020 to December 2020 were included in the current study. Six patients were excluded from this study due to meeting the exclusion criteria complicated infection and 3 patients refused to participate in this study. Finally, a total 426 patients were included in this study and their data was collected and analyzed (Figure 1). The information of basis clinical characteristics was obtained from the hospital database. The results showed that gender, age, educational level, CKD stage, Diabetes, weight loss and reduced food intake were related to SGA nutritional assessment (*P* < 0.05) (Table 1).

**Table 1:** Relationship between patients' general clinical characteristics and SGA nutritional assessment (*n*/%).

**Characteristic**		**SGA**		**χ^2^**	**Fisher exact test value**	***P*-value**
		**Normal**	**Malnutrition**			
Gender	Male	44 (18.5)	194 (81.5)	7.219		0.007
	Female	17 (9.0)	171 (91.0)			
Age, years	<40	12 (37.5)	20 (62.5)	31.123		0.001
	40~59	33 (22.1)	116 (77.9)			
	≥60	16 (6.5)	229 (93.5)			
Education level	<High school	39 (11.1)	313 (88.9)		17.776	0.001
	High school or equivalent	10 (27.0)	27 (73.0)			
	≥College	12 (32.4)	25 (67.6)			
Marital status	Married	55 (13.5)	351 (86.5)		5.836	0.054
	Unmarried/Divorce	6 (30.0)	14 (70.0)			
Payment	self-paid	11 (15.7)	59 (84.3)		2.278	0.517
	Rural cooperative medical system	3 (12.5)	21 (87.5)			
	Resident medical insurance	33 (12.7)	227 (87.3)			
	Employee medical insurance	14 (19.4)	58 (80.6)			
CKD stage	I/II	6 (42.9)	8 (57.1)		19.542	0.002
	III	16 (14.7)	93 (85.3)			
	IV	14 (10.3)	122 (89.7)			
	V	25 (15.0)	142 (85.0)			
Diabetes	No	51 (17.0)	249 (83.0)	5.941		0.015
	Yes	10 (7.9)	116 (92.1)			
Hypertension	No	22 (14.6)	129 (85.4)	0.012		0.913
	Yes	39 (14.2)	236 (85.8)			
Coronary heart disease	No	58 (15.5)	315 (84.5)	3.699		0.054
	Yes	3 (5.7)	50 (94.3)			
Gout	No	59 (14.5)	349 (85.5)	0.524		0.469
	Yes	2 (11.1)	16 (88.9)			
Cerebral infarction	No	59 (14.7)	342 (85.3)	0.404		0.353
	Yes	2 (8.0)	23 (92.0)			
Weight loss	No	60 (24.4)	186 (75.6)	48.130		0.001
	Yes	1 (0.6)	179 (99.4)			
Reduced food intake	No	59 (33.1)	119 (66.9)	88.334		0.001
	Yes	2 (0.8)	246 (99.2)			
Acute attack or complication of chronic disease	No	41 (15.0)	232 (85.0)	0.303		0.582
	Yes	20 (13.1)	133 (86.9)			
Long-term hemodialysis	No	59 (15.0)	334 (85.0)	1.326		0.250
	Yes	2 (6.1)	31 (93.9)			

CKD stage, Chronic Kidney Disease stage; SGA, Subjective Global Assessment.

Education level, marital status, payment and CKD stage were tested using Fisher Exact Test.

### Comparison of serum protein between malnutrition and normal nephropathy

Patients showed an incidence of malnutrition of 85.7%. The expression levels of serum ALB (31.91 ± 7.52 vs. 35.73 ± 5.72), PA (31.52 ± 9.05 vs. 36.44 ± 6.58) and Hb (91.20 ± 23.90 vs. 98.17 ± 27.69) in the malnutrition group were significantly lower than those in the normal group, and the difference was statistically significant (*P* < 0.05). There was no significant difference in serum TP (58.24 ± 9.82 vs. 60.12 ± 8.56) expression between the two groups (*P* > 0.05) (Table 2).

**Table 2:** Relationship between protein expression level and SGA nutritional assessment ($\bar{X}$ ± S).

**SGA**	***n***	**TP (60–83)**	**ALB (34–54)**	**PA (200–350)**	**Hb (115–150)**
Normal	61	60.12 ± 8.56	35.73 ± 5.72	36.44 ± 6.58	98.17 ± 27.69
Malnutrition	365	58.24 ± 9.82	31.91 ± 7.52	31.52 ± 9.05	91.20 ± 23.90
*T*		1.412	3.429	3.098	2.038
*P*		0.159	0.001	0.002	0.042
Mean difference		1.884	3.460	4.919	6.966
Degree of freedom		424	424	325	422
95% CI (Lower, Upper)		(−0.739, 4.508)	(1.477, 5.444)	(1.795, 8.042)	(0.248, 13.684)

SGA, Subjective Global Assessment; TP, total protein; ALB, albumin; PA, prealbumin; Hb, hemoglobin.

The normal value of health people was written closed with the indicators.

### Correlation analysis between SGA nutritional assessment and various protein expression levels in patients with CKD

The degree of malnutrition in patients with CKD was negatively correlated with the expression levels of serum ALB (*r* = −0.188), serum PA (*r* = −0.237) and Hb (*r* = −0.174) by using spearman correlation analysis (*P* < 0.05). There was no significant correlation between the degree of malnutrition and serum TP (*r* = −0.049) expression in patients with nephropathy (*P* > 0.05) (Table 3).

**Table 3:** Spearman correlation analysis between SGA nutritional assessment and protein expression levels in CKD patients Variables.

**Variables**		**ALB**	**PA**	**Hb**	**TP**
SGA	*r*	−0.188	−0.237	−0.174	−0.049
	*P*	0.001	0.001	0.001	0.312

SGA, Subjective Global Assessment; TP, total protein; ALB, albumin; PA, prealbumin; Hb, hemoglobin.

### Multivariate logistic regression analysis of influencing factors in patients with nephropathy due to malnutrition

**Figure 2:**
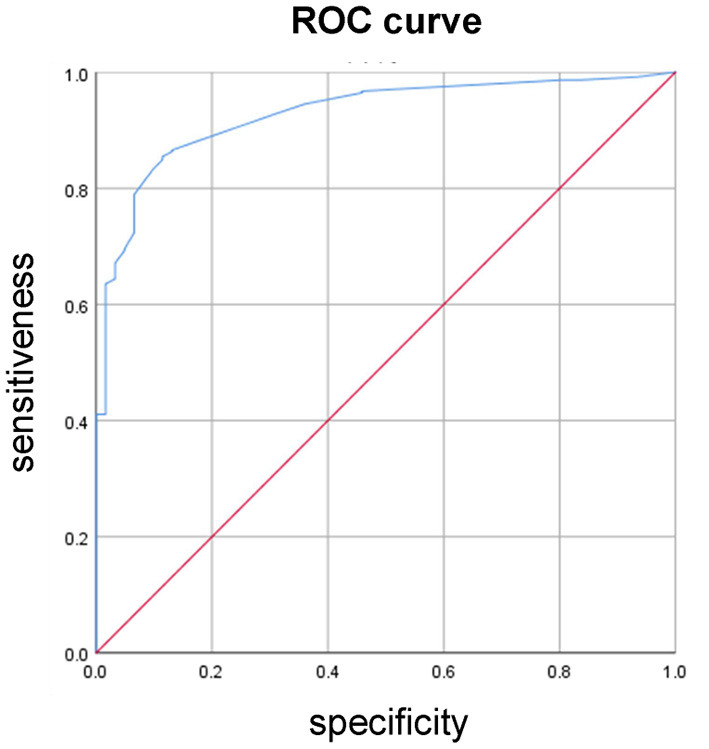
ROC curve.

According to the results of univariate analysis, a total of 5 variables were included in the multivariate Logistic regression analysis model according to the standard *P* < 0.10. The results showed that gender (OR = 2.371), age (OR = 3.646), weight loss (OR = 11.945), reduced food intake (OR = 27.888), serum albumin expression (OR = 2.936) were risk factors for malnutrition in patients with CKD, and the difference was statistically significant (*P* < 0.05) (Table 4). The Hosmer-Lemeshow test result, classification table result and area under the ROC curve (see Tables 5–7 and Figure 2).

**Table 4:** Analysis of related factors of malnutrition in patients with CKD.

**Factor**	**β**	**SE**	**Wald**	***P*-value**	**OR value**	**OR95% CI** **(Lower, Upper)**	**Co-linearity** **(tolerance)**	**Interactions** **(VIF)**
Gender	0.863	0.392	4.845	0.028	2.371	(1.099, 5.113)	0.982	1.018
Age, years	1.294	0.275	22.154	0.001	3.646	(2.127, 6.247)	0.978	1.023
Weight loss	2.480	1.088	5.194	0.023	11.945	(1,415, 100.811)	0.626	1.598
Reduced food intake	3.328	0.766	18.859	0.001	27.888	(6.210, 125.249)	0.620	1.613
ALB	1.077	0.301	12.772	0.001	2.936	(1.626, 5.299)	0.994	1.006
Constant	−5.054	1.030	24.065	0.001	0.006			

CKD stage, Chronic Kidney Disease stage; OR, odds ratio; VIF, variance inflation factor; Serum ALB level: <25 g/L is severe, 25–29 g/L is moderate, 30–35 g/L is mild. ALB, albumin.

The model assumption used here was binary logistic regress model. The co-linearity and interactions were analyzed by tolerance and VIF, respectively, there were no co-linearity and interactions.

**Table 5:** Hosmer-Lemeshow test result.

**χ^2^**	**Degree of freedom**	***P-*value**
4.656	8	0.794

**Table 6:** Classification table.

		**Predicted**		
		**SGA**		
**Actual**		**Normal**	**Malnutrition**	**Overall correctly classified percentage**
SGA	Normal	33	28	54.1
	Malnutrition	12	353	96.7
Total percentage				90.6

SGA, Subjective Global Assessment.

**Table 7:** Area under ROC curve.

**Area**	**SE**	***P*-value**	**95% CI** **(lower, upper)**
0.927	0.015	0.000	(0.897, 0.957)

### Correlation analysis between SGA nutritional assessment and urinary protein qualitative expression in patients with nephropathy

There was a positive correlation between SGA nutritional assessment and the qualitative expression of urinary protein using spearman correlation analysis (*P* < 0.05) (Table 8).

**Table 8:** Spearman correlation analysis between SGA nutritional assessment and urinary protein in CKD patients.

**Variables**	**Urinary protein**	
SGA	*r*	0.268
	*P-*value	0.000

## Discussion

CKD is a global health problem (9). Poor nutritional status is a serious consequence of patients with CKD and a strong indicator of adverse outcome of chronic kidney disease (10). Malnutrition is associated with a variety of changes such as metabolic acidosis, altered intestinal flora, and hormonal dysregulation, all of which may contribute to the progression of kidney disease and increase morbidity and mortality (11). A recent study also pointed out that malnutrition is associated with the excessive daytime sleepiness in older patients with CKD, which is related with higher risks of morbidity and mortality. The excessive daytime sleepiness and nutritional status should be evaluated together in clinical practice (12). Besides the prevalence of malnutrition has been reported in various studies to be between 20 and 75%, depending on the diagnostic criteria and the subjects studied. The incidence of malnutrition in hospitalized patients with CKD was between 20 and 60% (13). Especially in the late stage of CKD, the incidence of malnutrition ranges from 23 to 75% (14). Ultimately, the consensus reached by scholars is that the prevalence of malnutrition in CKD patients is at a high level.

SGA is a comprehensive nutritional status assessment tool proposed by Detsky et al. (15) in 1987. It is one of the commonly used nutritional evaluation methods in clinical practice and is used to detect the prevalence of malnutrition and determine its prognosis (16). The study of Kopple et al. confirmed that the incidence of malnutrition gradually increased with the progression of renal insufficiency. Therefore, when CKD is in stage 3–5, nutritional screening and intervention should be performed routinely (17, 18). In this study, patients were divided into normal group and malnourished group according to SGA score results, and there were statistically significant differences in the results of hemoglobin, ALB and PA in biochemical examination indicators of CKD patients in the two groups.

In our multivariate logistic regression model, gender, age, weight loss, reduced food intake, and serum ALB expression were independently associated with malnutrition in patients with chronic kidney disease. On the contrary, H.K. Aggarwal et al.'s study pointed out that gender is not related to malnutrition (19), which may be related to the included study subjects and the evaluation criteria of malnutrition.

Age is a risk factor for mortality in maintenance hemodialysis patients and is also one of the major risk factors for malnutrition (20). With the increasing age of CKD patients and the progression of disease, the gastrointestinal function is abnormal, the function of various organs of the body decreases, and the absorption and metabolism rate slows down, leading to insufficient protein and energy intake and aggravating malnutrition, thus leading to poor prognosis.

Plasma ALB is the most important indicator of protein storage in the body and the gold standard for evaluating nutritional status (11). In this study, serum ALB, PA and hemoglobin in the malnourished group were lower than those in the non-malnourished group, suggesting that the Hb level of patients was closely related to their nutritional status, and consistent with BMI, ALB, and other indicators reflecting the nutritional status of patients. Therefore, for patients with reduced BMI, relevant biochemical indicators should be detected in time to improve malnutrition. Protein catabolism and nitrogen balance in CKD patients are closely related to energy intake, and negative energy intake will accelerate protein catabolism and make protein be utilized from energy supply, leading to negative nitrogen balance (11). Inadequate nutrient intake is common in CKD and ESRD populations and poses a direct risk of protein malnutrition. In addition, the resting energy consumption of CKD patients is higher than that of non-CKD patients (21), so CKD patients should pay attention to energy and nutritional supplement. Siren Sezer et al. showed a significant increase in serum albumin following long-term oral nutritional supplements and a significant decrease in the percentage of patients with serum ALB <3.5 g/dL at the end of the follow-up period. These findings suggest that long-term oral nutritional supplements can improve the prognosis of malnutrition in CKD (22, 23).

Qualitative examination of urinary protein is a reflection of the loss of protein in routine urine. The more urinary protein loss, the higher the incidence of malnutrition in patients, which is basically consistent with the relevant literature (24). In the past, nephrologists' understanding of the harm of urinary protein mainly focused on the fact that it can lead to the progression of CKD, increase the risk of cardiovascular disease, and increase all-cause mortality (25). There are few reports on the relationship between urinary protein and nutritional risk by searching the literature. We speculate that the possible mechanisms include: protein loss from the urine will lead to hypoproteinemia, and protein loss is often accompanied by the loss of other nutrients, which will directly lead to malnutrition; In addition, urinary protein can also cause oxidative stress and micro inflammation, and indirectly lead to malnutrition by interfering with human nutrition metabolism. This suggests that we should pay attention to the nutritional risk of patients with urinary protein besides reducing urinary protein by drugs. Appropriate nutritional support can also improve the prognosis. However, the specific mechanism remains to be further explored.

Limitations, firstly, in the current study, the numbers of total patients was not big enough and the patients with normal SGA assessment was much less than malnutrition SGA patients, more cases should be included to expand and strong this research conclusion. Secondly, the SGA assessment was only divided into two classification normal and malnutrition, a further study using more grades of SGA assessment should be considered.

## Conclusion

In conclusion, nutritional status is an important factor affecting the prognosis of patients with CKD, and it is of great significance to evaluate and improve the nutritional status of patients to improve the quality of life of patients and reduce mortality. We should conduct dynamic assessment of the nutritional status of patients with CKD and timely intervention to delay the progression of chronic kidney disease. It is better to develop personalized diet plan for each patient under the guidance of nephrology nutritionists and with the joint participation of nephrology physicians and nephrology nurses. However, this study was only a cross-sectional study, and further cohort studies are needed in the future to observe the effects of nutritional intervention on the progression and prognosis of chronic kidney disease.

## Data availability statement

The original contributions presented in the study are included in the article/supplementary material, further inquiries can be directed to the corresponding author.

## Ethics statement

The studies involving human participants were reviewed and approved by Ethics Committee of the First Affilated Hospital of Wannan Medical College. The patients/participants provided their written informed consent to participate in this study.

## Author contributions

Conception and design of the research: W-zX and CW. Acquisition of data: W-zX, CW, and Y-lL. Analysis and interpretation of the data: L-LW and Y-hC. Statistical analysis: W-zX, CW, Y-lL, L-LW, and Y-hC. Writing of the manuscript: W-zX and Y-lL. Critical revision of the manuscript for intellectual content: W-zX. All authors contributed to the article and approved the submitted version.
